# Importance of four-chamber assessment using open-window mapping in posterior septal accessory pathways: A case report

**DOI:** 10.1016/j.hrcr.2025.07.014

**Published:** 2025-07-24

**Authors:** Shinichi Harada, Masato Okada, Koji Tanaka, Yusuke Ikada, Nobuaki Tanaka

**Affiliations:** 1 Cardiovascular Center, Sakurabashi Watanabe Advanced Healthcare Hospital, Osaka, Japan; 2 Cardiovascular Division, Mie University Hospital, Tsu, Japan

**Keywords:** Accessory pathway, Catheter ablation, Electroanatomic mapping, Open-window mapping, Wolff–Parkinson–White syndrome

**Key Teaching Points**

• Posterior septal accessory pathways (APs) are located within the complex inferior pyramidal space involving 4 cardiac chambers and the coronary sinus. They occasionally traverse both the left and right myocardium.
• During orthodromic reciprocating tachycardia, the atrial, ventricular, and AP potentials are often broadly fused around such APs, producing multicomponent continuous electrograms that hinder a precise annotation of the atrial and ventricular signals.
• Open-window mapping enables visualization of conduction gaps without requiring separation or annotation of fused signals.
• Given the anatomic complexity of the cardiac crux region, meticulous and repeated 4-chamber mapping is essential for successful ablation of posterior septal APs.

## Introduction

Open-window mapping (OWM) is a novel automated mapping method for catheter ablation of accessory pathways (APs). This method expands the window of interest to analyze both atrial and ventricular signals simultaneously. When combined with the extended early-meets-late (EEML) algorithm, OWM facilitates the visualization of the location, width, and conduction pathway of APs in patients with Wolff–Parkinson–White syndrome.^1^, ^2^, ^3^, ^4^, ^5^ However, its utility in cases of posterior septal APs, particularly those that traverse both the left and right myocardium, has rarely been reported. Here, we describe a case of a broad posterior septal AP that required repeated OWM across all 4 chambers to identify both atrial and ventricular insertion sites. This case illustrates not only the potential advantages of OWM but also its limitations in anatomically complex pathways, emphasizing the importance of repeated 4-chamber assessment to guide successful ablation.

## Case report

**Figure 1:**
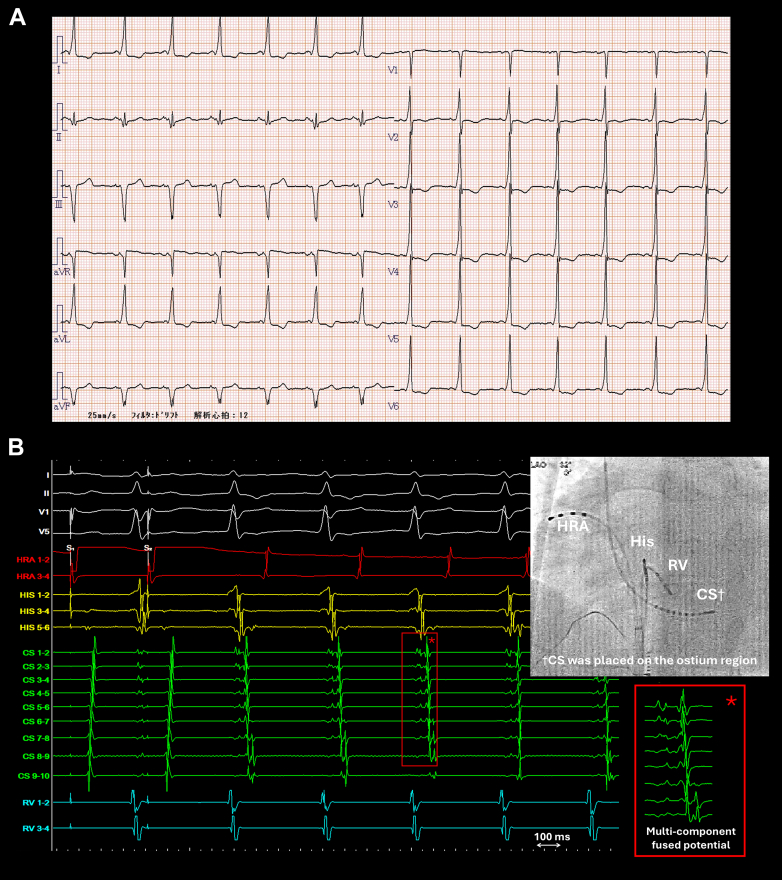
**A:** The 12-lead electrograms during sinus rhythm. **B:** The intracardiac electrograms and fluoroscopic image during the initiation of orthodromic reciprocating tachycardia. Multicomponent fused electrograms (asterisk) were recorded across a broad range of the CS electrodes. I, II, V1, and V5 represent surface electrocardiogram; His 1–2 and 5–6 represent distal to proximal His catheter electrograms; CS 1–2 to 9–10 represent distal to proximal CS recordings; and RV 1-2 and 3-4 represent distal to proximal right ventricular recordings. AP = accessory pathway; CS = coronary sinus; His = His bundle; HRA = high right atrium; LAO = left anterior oblique view; RV = right ventricle.

A 24-year-old woman without a history of heart disease was referred to our hospital for evaluation of intermittent presyncope. The 12-lead electrocardiogram revealed delta waves, consistent with manifest Wolff–Parkinson–White syndrome (Figure 1A). The AP was presumed to be located on the right posterior septum. Holter electrocardiographic monitoring documented a regular narrow QRS complex tachycardia during presyncope episodes, suggesting orthodromic reciprocating tachycardia (ORT) as the underlying mechanism.

After obtaining an informed consent, an electrophysiological study was performed under local anesthesia. Electrode catheters were positioned in the right atrial appendage, His bundle region, coronary sinus (CS), and right ventricular apex. Delta waves were intermittently present, and when observed, the His potentials overlapped with the ventricular potentials, with a His–ventricular interval of −20 ms. Ventricular pacing demonstrated ventriculoatrial (VA) conduction, with the earliest atrial activation recorded at the proximal CS. Ventricular extrastimuli did not exhibit decremental conduction, suggesting that the VA conduction was via the AP. In contrast, in the absence of delta waves, atrial extrastimuli revealed decremental conduction without evidence of a jump-up phenomenon. Fortunately, a regular narrow QRS tachycardia with a cycle length of 385 ms was induced by programmed stimulation (basic cycle length 600 ms, S1S2 380 ms) (Figure 1B). The atrial activation sequence was identical to that observed during ventricular pacing. A single right ventricular stimulus during the His-refractory period reset the tachycardia. Ventricular overdrive pacing terminated the tachycardia, and atrial resetting was observed during the fusion period, confirming the diagnosis of ORT via posterior AP.

**Figure 2:**
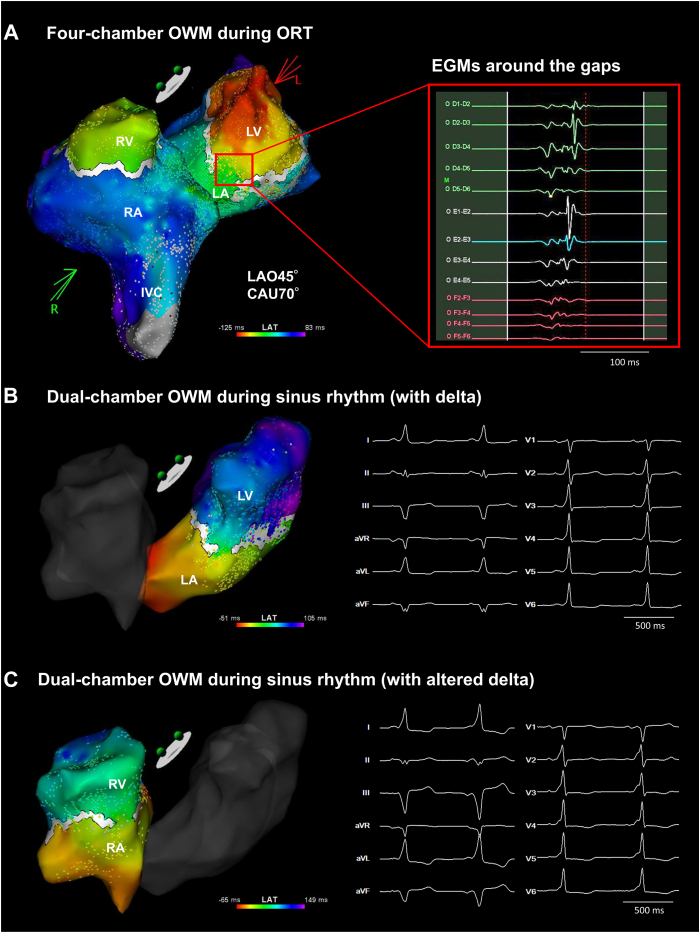
**A:** Four-chamber OWM during ORT, created with the CARTO 3 system and Octaray catheter. Ventriculoatrial conduction was evaluated using an automated wavefront annotation. A sufficient window of interest encompassing both atrial and ventricular signals was applied. The *white line* indicates a local conduction block identified by the EEML algorithm. The lower threshold was initially set at 25% and manually adjusted to 28% to visually align the conduction gap with the *yellow-to-green transition zone* on the propagation map (Supplemental Movie 1). A conduction breakthrough was identified on the posterior septum within the left ventricle, and multicomponent fused electrograms were observed at that site. **B:** The left-sided dual-chamber OWM during sinus rhythm with manifest delta waves (Supplemental Movie 2, left panel). The EEML lower threshold was set to 30% using the same alignment method. The earliest ventricular activation site corresponded to the gap in the *white line*. The AP–ventricular junction was located near the previous ablation site on the left posterior septum. An additional radiofrequency application at that site slightly altered the delta wave morphology. **C:** The right-sided dual-chamber OWM during sinus rhythm with altered delta waves (Supplemental Movie 2, right panel). The lower threshold was adjusted to 22%. A second AP–ventricular junction was identified on the right posterior septum. The earliest ventricular activation site again matched the gap in the *white line*. A radiofrequency application at that site induced a junctional rhythm and ultimately eliminated the delta waves. EEML = extended early-meets-late; IVC = inferior vena cava; LA = left atrium; LV = left ventricle; ORT = orthodromic reciprocating tachycardia; OWM = open-window mapping; RA = right atrium. Other abbreviations are as in Figure 1.

We initially mapped the right atrium during ORT and observed that the fused, continuous potentials in the CS preceded any right atrial signals. We estimated that the latter component represented the earliest atrial activation and attempted ablation at that site. However, a high impedance, unstable contact force, and severe patient discomfort precluded continuation of ablation at that site. To improve localization, OWM was performed in the right and then in the left heart during tachycardia using the CARTO 3 system and an Octaray catheter (Biosense Webster, Irvine, CA). The lower threshold of the EEML algorithm was initially set at 25% and subsequently adjusted to achieve visual alignment between the white line (local conduction block) and the yellow-to-green transition zone (conduction gap) on the propagation map. Upon increasing the lower threshold to 28%, a discrete conduction gap became evident along the posterior mitral annulus (MA), consistent with the localization of a left posterior septal AP (Figure 2A) (Supplemental Movie 1). Radiofrequency (RF) energy was then delivered at that site using a ThermoCool SmartTouch SF catheter (Biosense Webster) (30 W for 30 seconds), which successfully terminated the tachycardia and diminished the delta waves. However, after the administration of isoproterenol, the delta waves reappeared, indicating residual preexcitation.

To identify the AP–ventricular junction, OWM was repeated during sinus rhythm (Figure 2B) (Supplemental Movie 2, right panel). The earliest ventricular activation site was located on the left posterior septum, close to the previous ablation site. Additional RF energy applications at that site did not significantly alter the local electrograms; however, the QRS complex became wider and the delta waves in the inferior leads deepened, suggesting modification of the residual AP conduction at that site.

Because VA conduction exhibited decremental properties and no continuous electrograms were recorded along the MA, OWM was subsequently performed in the right heart during sinus rhythm with altered delta waves. The EEML tool revealed a conduction gap in the white line at the 5–6-o’clock position on the tricuspid annulus (TA) (Figure 2C) (Supplemental Movie 2, left panel). Given the proximity of this septal site to the atrioventricular (AV) node, RF energy was titrated from 10 W to 30 W. Ablation at the posterior septal region (5 o’clock on the TA) induced a junctional rhythm but ultimately eliminated the delta waves. Subsequent ventricular extrastimulation demonstrated clear decremental VA conduction, indicating retrograde conduction through the AV node. Postprocedural electrophysiological studies confirmed decremental AV conduction without evidence of residual AP conduction. The patient was discharged without antiarrhythmic medications and has remained free of presyncope at 1-year follow-up.

## Discussion

Catheter ablation of posterior septal APs is challenging owing to their complex anatomic location and variable conduction properties. These pathways often span multiple chambers and may traverse epicardial structures such as the middle cardiac vein or its diverticulum.^6^ The intricate 3-dimensional architecture of the crux region complicates catheter access, stability, and electrogram interpretation. In this case, the need for repeated OWM from both the left and right chambers highlighted the difficulty in fully delineating such pathways using a single map.

**Figure 3:**
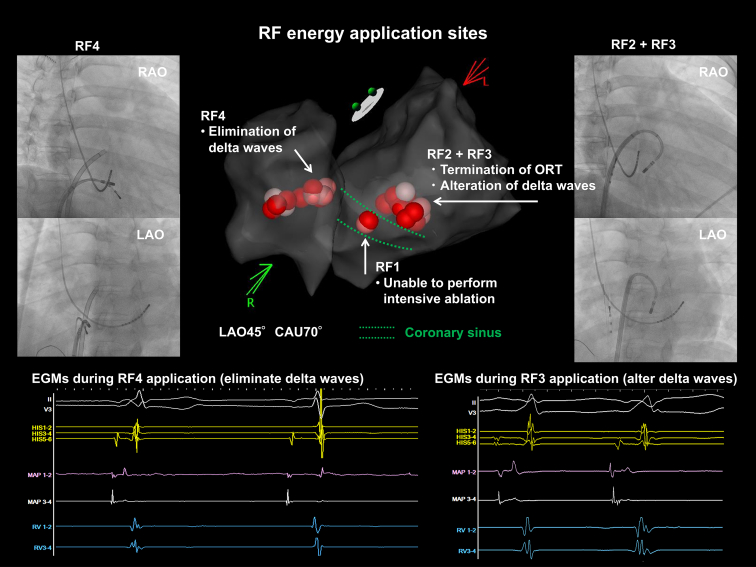
Electroanatomic maps, fluoroscopic images, and intracardiac electrograms at the RF energy application sites (from RF1 to RF4). RF energy was initially applied at the earliest activation site within the coronary sinus (RF1 site), but could not be continued owing to high impedance, instability, and pain. OWM during the tachycardia revealed the AP–atrial junction at the RF2 site, and an RF energy application at that site successfully terminated the tachycardia. OWM during sinus rhythm demonstrated 2 AP–ventricular junctions, identified sequentially: first at the left posterior septum (RF3 site) and then at the right posterior septum (RF4 site). Note: Fluoroscopic images were retrospectively captured, and the His catheter was not visible in the imaging field. EGM = electrogram; OWM = open-window mapping; RAO = right anterior oblique view; RF = radiofrequency. Other abbreviations are as in Figure 1.

As illustrated in Figure 3, RF energy was broadly applied around the initial ablation site where AP potentials were recorded (RF1). In a stepwise manner, RF energy applications to the septal MA (RF2) terminated the ORT, whereas additional applications near the previous ablation site (RF3) altered the delta waves. Subsequent RF energy applications to the septal TA (RF4) successfully eliminated the residual preexcitation. Although 4-chamber OWM during ORT revealed a single gap on the posterior septal MA, successful elimination of the delta wave required additional ablation on both sides. Although the presence of 2 distinct APs could not be entirely ruled out, the sequential electrophysiological responses to ablations were more consistent with a single broad AP exhibiting dual ventricular insertions. In cases with posterior septal APs, conduction characteristics may vary between AP segments, depending on their anatomic course and myocardial insertion. These observations underscore the importance of comprehensive and repeated mapping for the successful ablation of complex posterior septal APs.

Certainly, OWM with the EEML algorithm has non-negligible limitations, given that the lower threshold of the EEML algorithm significantly influences the visualization of the conduction gaps. If the threshold is set too low, it may create false conduction block; if set too high, it may miss narrow conducting gaps. In our case, the threshold was adjusted to align the gap with the transition zone based on the propagation map, which may have included an arbitrary aspect but was consistent with the previous report, featuring a yellow-green transition.^2^ Nevertheless, when appropriately optimized, these tools provide more accurate visualization of the AP location, along with reduced fluoroscopy time, RF time, and overall mapping time, than conventional point-by-point mapping.^3^ An additional practical advantage of OWM lies in its ability to interpret fused electrograms without requiring precise separation of the atrial, ventricular, and AP potentials. In many posterior septal APs, these signals are closely spaced or even fused, making it challenging to clearly annotate the individual signals. OWM addresses this limitation by visualizing global activation patterns, enabling the effective delineation of AP conduction based on composite potentials. In the present case, the tools accurately localized the AP–atrial junction during ORT and the AP–ventricular junction during sinus rhythm. Nonetheless, the inherent anatomic complexity and variable conduction properties of the posterior septal APs necessitate individual assessment of both VA and AV conduction. Repeated OWM was necessary to achieve the complete elimination of such APs.

Several limitations and precautions should be acknowledged when using OWM and the EEML algorithms to identify posterior septal APs. First, some posterior septal APs exhibit decremental conduction properties, and the utility of OWM in such cases remains uncertain. Second, posterior APs may extend into the coronary venous system, but the use of multielectrode catheters with small and closely spaced electrodes within the coronary venous branches is not feasible. Finally, identifying a conduction gap on the white line does not guarantee procedural safety. As demonstrated in this case, an RF application to the posterior septal region induced a junctional rhythm, suggesting the proximity to the AV node. Careful consideration of procedural safety is essential before delivering RF energy in this anatomically and physiologically sensitive region.

## Conclusion

OWM and the EEML algorithm are valuable tools for identifying the location, width, and propagation paths of posterior septal APs. However, owing to the complexity of the inferior pyramidal space involving 4 chambers and the coronary venous system, meticulous and repeated 4-chamber assessment is essential for successful ablation.

## Disclosures

The authors have no conflicts of interest to disclose.

## References

[1] Schricker A.A., Winkle R., Moskovitz R. (2021). Open-window mapping of accessory pathways utilizing high-density mapping. J Interv Card Electrophysiol.

[2] Wang N.C. (2022). Open-window mapping and the extended early-meets-late algorithm for the Wolff-Parkinson-White syndrome. J Arrhythm.

[3] Yagishita A., Yamauchi Y., Sagawa Y. (2023). Utility of open-window mapping for catheter ablation of an accessory pathway in patients with Wolff-Parkinson-White syndrome. Pacing Clin Electrophysiol.

[4] Dulai R., Bangash F., Sharma A. (2023). Open window mapping of accessory pathways: A literature review and practical guide. Arrhythm Electrophysiol Rev.

[5] Sande J.L.M., Minguito-Carazo C., Melchor L.G. (2025). Open window mapping with extended early meets late algorithm vs. conventional mapping for accessory pathway ablation. J Interv Card Electrophysiol.

[6] Macedo P.G., Patel S.M., Bisco S.E., Asirvatham S.J. (2010). Septal accessory pathway: anatomy, causes for difficulty, and an approach to ablation. Indian Pacing Electrophysiol J.

