# Supplementary material for: Importance of four-chamber assessment using open-window mapping in posterior septal accessory pathways: A case report
Source: HeartRhythm Case Rep. 2025 Jul 24;11(10):1038–42. doi: 10.1016/j.hrcr.2025.07.014 (PMC12666908; doi:10.1016/j.hrcr.2025.07.014)
Supplement: Supplementary Material [file mmc3.docx]

**Supplemental Movie 1 legend**

The dynamic four-chamber propagation image during orthodromic reciprocating tachycardia. Left- and right-sided open-window mappings (OWMs) were individually acquired and subsequently integrated using a common electrode set as a reference. The white line denotes a local conduction block, visualized using the extended early-meets-late algorithm with a manually adjusted lower threshold of 28%.

**Supplemental Movie 2 legend**

The dynamic dual-chamber propagation images during sinus rhythm with manifest delta waves. The left and right panels show the right-sided and left-sided dual-chamber OWMs, respectively. The white line denotes a local conduction block, visualized using the extended early-meets-late algorithm with a manually adjusted lower threshold of 22% in the right heart and 30% in the left heart, respectively.
